# Genome-Wide Identification of the *SRS* Gene Family in Cucurbitaceae: Clade Identification and Expression Analysis of *CmSRS* Genes Under Drought and Salt Stress

**DOI:** 10.3390/biology14070891

**Published:** 2025-07-20

**Authors:** Haozhe Min, Kexiang Wang, Yao Guo, Junyan Yang, Xuhui Wang, Miao He, Tao Lin, Jiancai Mao, Zhengying Xuan

**Affiliations:** 1 Department of Horticulture, College of Horticulture and Forestry, Tarim University, Aral 843300, China; mhz13319903112@163.com (H.M.); 18160256643@163.com (M.H.); 13592878760@163.com (T.L.); 2 Fruit and Vegetable Research Institute, Xinjiang Academy of Agricultural Sciences, Urumqi 830091, China; 13289933973@163.com (Y.G.); yangjunyan@xaas.ac.cn (J.Y.); 13123150031@163.com (X.W.); 3 Xinjiang Production & Construction Corps Key Laboratory of Protected Agriculture, Aral 843300, China; 4 Hami Agricultural and Animal Husbandry Industry Development Investment Co., Hami 839000, China; laowang.110@163.com; 5 Department of Horticulture, Faculty of Agriculture, Shihezi University, Shihezi 832000, China

**Keywords:** Cucurbitaceae, melon, *SRS*, bioinformatics, adversity stress

## Abstract

Background: The short strand-related sequence (SRS) gene family is a class of plant-specific transcription factors related to a group of genes known as the short internode (SHI) or SRS/STY gene family, which plays important roles in regulating plant growth and development and stress responses. Although the *SRS* genes have been studied in many plants, in cucurbit crops, they have thus far only been identified in cucumber. Methods: In the Cucurbitaceae database from melon (*Cucumis melo*), cucumber (*Cucumis sativus*), watermelon (*Citrullus lanatus*), bottle gourd (*Lagenaria siceraria*), wax gourd (*Benincasa hispida*), moschata pumpkin (*Cucurbita moschata)*, and pumpkin (*Cucurbita maxima*), a total of 60 *SRS* genes were identified in seven Cucurbitaceae crops, which were classified into three subfamilies. Results: The same subfamily showed conserved motifs and gene structures. The differences in the number of *SRS* genes in different Cucurbitaceae crops implied likely gene loss or duplication events during evolution. Analysis of promoter *cis*-regulatory elements indicated that these *SRS* genes may be involved in hormone response, growth and development, and biotic and abiotic stress responses in plants. Most of the *CmSRS* genes in melons were expressed in the roots, with a few expressed in the leaves and ovaries. In addition, *CmSRS* expression was induced by biotic (wilt and powdery mildew) and abiotic (drought and salt) stresses. Subcellular localization of CmSRS proteins showed predominant expression in the nucleus. Conclusions: A total of 60 Cucurbitaceae *SRS* genes are present in the genomes of seven Cucurbitaceae crops. These cucurbit *SRS* genes seem to have maintained similar characteristics and functions during the evolutionary process. These results lay the foundation for the study of biological functions of *SRS* genes in Cucurbitaceae crops.

## Simple Summary

SRS, a transcription factor unique to plants, plays important roles in regulating plant growth and development and in responding to stress. Although *SRS* genes have been studied in many plants, in cucurbit crops, they have only been identified in cucumber thus far. This study not only presents a comprehensive bioinformatics examination of all *SRS* genes in the genomes of seven Cucurbitaceae crops, but also investigates the expression levels of *CmSRS* genes in different tissues. In addition, the expression of *CmSRS* genes under abiotic (drought and salt) and biotic (wilt and powdery mildew) stresses, as well as their subcellular localization, are analysed. These results lay the foundation for studying the biological functions of *SRS* genes in Cucurbitaceae crops.

## 1. Introduction

The SHI-related sequence (SRS) gene family is a family of genes related to plant-specific transcription factors, also known as the short internode (SHI) or SRS/STY family, consisting of two highly conserved structural domains, RING and IXGH [1]. The RING domain is located at the N-terminal end of a CH3CH3 motif-containing RING zinc finger structure (CX2CX7CX4CX2C2X6C) [2], which was initially identified as a DNA-binding motif in the African clawed frog [3]. In plant cells, the RING domain binds to RNA, proteins, and lipid substrates and is involved in a variety of physiological and biochemical processes [4]. The IXGH domain, located at the C-terminal end, contains acidic amino acids and is homodimerized [5], a trait which has not been found in other proteins and may be a unique feature of the *SRS* family [6].

The SRS protein family plays key roles in a variety of physiological and biochemical processes in plants, including hormone synthesis and signal transduction, abiotic stress response, and plant organ growth and development [7]. The *SRS* family is widely distributed in plants. In *Arabidopsis thaliana*, a total of 10 *SRS* genes were identified, including *SHI, STY1, STY2, LPR1*, and *SRS3/4/5/6/7/8*. Except for the *SRS8* gene missing the IXGH structural domain, the remaining nine genes contain both the RING structural domain and the IXGH structural domain [7,8,9,10]. Functionally, *STY1* (*SRS1*) plays an important role in the development of apical meristematic tissue auxin biosynthesis [11]. LPR1 is able to form protein complexes with SHI, STY1, SRS3, SRS6, and SRS7, which regulate lateral root development by modulating auxin signalling and chromatin modification [8,12,13]. However, *LPR1* overexpression inhibits root development, which is mainly due to elevated auxin levels [14]. Overexpression of *AtSHI* in *Arabidopsis thaliana*, *Longevity Flower*, and *Pongamia monnieri* showed a dwarfing phenotype. These studies suggest that *AtSHI*, as a negative regulator in gibberellin (GA) signalling, affects plant stature by regulating stem elongation [5,15,16]. Meanwhile, in rice (*Oryza sativa* L.), *OsSHI* increases tiller number and reduces spike size by regulating the transcriptional activity of IPA1 (ideal plant architecture 1) [17].

*SRS* genes may function as key regulators in plant abiotic stress response. It has been shown that the 9 *MaSRS* genes of *Melilotus albus* exhibited significant up- and downregulated expression at different time points under salt, low temperature, salicylic acid (SA) and methyl jasmonate (MeJA) treatments [18]. In soybean, *GmSRS18* has been identified as a key gene for negatively regulating drought and salt tolerance [1]. In cotton, *GhSRS21* is also involved in salt stress response as a negative regulator [19]. However, in cucurbit crops, the identification of *SRS* genes and their expression patterns have been reported only in cucumber, despite the increasing research on the function of *SRS* genes in various plants.

Cucurbitaceae crops are widely distributed in subtropical and tropical regions and play critical roles in global economic development [20]. These crops are rich in important nutrients [21,22], exhibit medicinal value in promoting cardiovascular health [23,24], and are used in the treatment of many diseases [25,26]. Among them, melon (*Cucumis melo* L.) is one of the major cash crops in China [27]. According to statistics, the global annual production of melon is estimated to be more than 40 million tonnes [28]. However, its final yield is often affected by pests, diseases, and abiotic stresses [29]. In particular, abiotic factors such as high temperatures, high salinity, and drought can lead to an average yield reduction of about 50% in crops [30]. In order to gain a deeper understanding of the functions of *SRS* genes in Cucurbitaceae crops, the present study was carried out to systematically analyse seven cucurbit crops. A total of 60 *SRS* genes were identified, and their gene structures, conserved motifs, conserved structural domains, chromosomal localization, cis-acting elements, and phylogenies were comprehensively analysed. The covariance relationship between the *SRS* genes of melon and six other Cucurbitaceae crops was also explored. Finally, the expression pattern of the melon *CmSRS* gene under *Fusarium acnes* and *Fusarium* powdery mildew infestation was analysed using transcriptome data. The expression characteristics of the *CmSRS* gene in different tissues, as well as under drought stress, were analysed by qRT-PCR. This study aims to provide a theoretical basis for the functional identification of *SRS* genes in cucurbit crops, as well as an important reference for further studies related to *SRS* genes and melon growth, development, disease resistance, and abiotic stress tolerance.

## 2. Materials and Methods

### 2.1. Identification of SRS Genes in Cucurbitaceae

The HMM model file (PF05142) for the *SRS* family was downloaded from the Pfam database (http: //pfam.xfam.org/ (accessed on 15 September 2024)). Protein sequences, genomes, and annotation files for seven cucurbit crops were obtained from the Cucurbitaceae database (http://www.cucurbitgenomics.org/ (accessed on 15 September 2024)), while protein sequences, genomes, and annotation files for *Arabidopsis thaliana* and rice were downloaded from the Ensemble Plants database (https://plants.ensembl.org/ (accessed on 15 September 2024)). Protein sequences were extracted using Fasta Extract (Recommended) plug-in of Tbtools. *SRS* gene family members were predicted in the genomes of seven Cucurbitaceae crops, *Arabidopsis thaliana,* and rice using the Simple HMM Search plug-in in TBtools v2.300 software [31]. Candidate *SRS* genes were identified using the NCBI-Conserved Domain Database (CDD) (http: //www.ncbi.nlm.nih.gov/Structure/cdd/wrpsb.cgi (accessed on 15 September 2024)).

### 2.2. Physicochemical Traits and Chromosomal Distribution of SRS in Cucurbitaceae

Physicochemical properties such as amino acid number, molecular weight, and isoelectric point of *SRS* family members of seven cucurbit crops were analysed using the ExPASy online software (https://web.expasy.org/protparam/ (accessed on 15 September 2024)). Chromosome physical mapping of *SRS* family members in seven Cucurbitaceae crops was performed using the mg2c online software (http://mg2c.iask.in/mg2c_v2.1/ (accessed on 15 September 2024)).

### 2.3. Analysis of Gene Structure, Conserved Motifs, and Evolution of the SRS Gene in Cucurbitaceae

The One Step Build an ML Tree plug-in and Simple MEME Wrapper v2.300 plug-in of Tbtools software were used for the *SRS* gene evolutionary analysis and motif analysis of Cucurbitaceae, respectively. The Gene Structure View v2.300 (Advanced) plug-in was used to merge and visualise the results. Phylogenetic analysis was performed using MEGA11, and the evolutionary tree was constructed using the neighbour-joining method [32]. The evolutionary tree was presented using the evolview online tool (https://evolgenius.info//evolview-v2/ (accessed on 15 September 2024)).

### 2.4. Analysis of Covariance of SRS Genes in Cucurbitaceae

Covariance analyses were performed for each of the seven Cucurbitaceae crops, *Arabidopsis thaliana,* and rice species using the One Step MCScanX plug-in v2.300 of Tbtools software. The results were combined for visualisation using the Multiple Synteny Plot plug-in v2.300.

### 2.5. Analysis of the Role of Cis-Elements in the Promoter of the SRS Genes in Cucurbitaceae

The GXF Sequences Extract plug-in v2.300 of Tbtools software was used to extract 2000 bp promoter sequences upstream of the transcription start site. These promoter sequences were submitted to the PlantCARE online analysis tool (https://bioinformatics.psb.ugent.be/webtools/plantcare/html/ (accessed on 16 September 2024)) for *cis*-acting element prediction. Heat mapping was performed using the Heat Map plug-in v2.300.

### 2.6. RNA Extraction and qRT-PCR Analysis

In order to analyse the expression pattern of *CmSRS* genes in various organ tissues and the expression changes under drought and salt stress, RNA was extracted using the TIANGEN Plant Total RNA Extraction Kit (Beijing, China). cDNA was reverse-transcribed from quality-tested RNA using the TIANGEN FastKing One-Step De-genomic cDNA First Strand Synthesis Kit (Beijing, China). qRT-PCR was performed using the melon *ACTIN* gene (GenBank: AY859055.1) as an internal reference. The reactions were carried out using the SGExcel FastSYBR qPCR kit (Shanghai, China) on a 96-well reaction plate with cDNA as the template. The qRT-PCR reaction system consisted of 1 μL of 2× SGExcel FastSYBR Mixture, 1 μL of cDNA template, 0.4 μL each of forward and reverse primers, as well as ddH_2_O to a final volume of 20 μL. The PCR amplification program was as follows: 95 °C for 3 min, followed by 95 °C for 5 s and 60 °C for 20 s. The relative expression of the gene was calculated using the 2^−△△Ct^ method. To ensure the accuracy and reliability of the experimental results, three biological replicates were included for each sample, and each PCR reaction was performed with three technical replicates. The primers are listed in Supplemental Table S1.

### 2.7. Transcriptome Data Analysis

Transcriptome sequencing data PRJEB15551 and PRJNA434538 from the NCBI database were used to analyse the expression of melon *SRS* genes under Fusarium spinosum and powdery mildew fungus infestation. Heat maps were drawn using TBtools.

### 2.8. Subcellular Localization

The fusion proteins were constructed by cloning the coding sequences of *CmSRS1*, *CmSRS3*, and *CmSRS4,* without the stop codon, into a Super1300 vector containing green fluorescent protein (GFP). Then, the fusion plasmid was transformed into *Agrobacterium tumefaciens* (GV3101). Fused CmSRS-GFP proteins were transiently expressed in 4-week-old *Nicotiana benthamiana* leaves. The GFP fluorescence signal was analysed by confocal microscopy. The primers are listed in Supplemental Table S2.

### 2.9. Interaction Network and Protein Structure Prediction of Melon SRS Proteins

Protein interaction network prediction was performed using the STRING database (STRING functional protein association networks (https://cn.string-db.org/cgi/input?sessionId=bXV51TlyIO7u&input_page_show_search=on (accessed on 17 September 2024)). The online website SOPMA (https://npsa-prabi.ibcp.fr/cgi-bin/npsa_automat.pl?page=npsa_sopma.html (accessed on 17 September 2024)) was used for protein secondary structure prediction. The prediction of the protein tertiary structure was completed using the SWISS-MODEL online website (http://swissmodel.expasy.org/ (accessed on 17 September 2024)).

## 3. Results

### 3.1. Identification and Physicochemical Properties of Seven SRS Genes in Cucurbitaceae

Using the HMM Search tool in Tbtools software, a total of 60 *SRS* family members were identified in the whole genomes of seven Cucurbitaceae crops in this study (Table 1), with the following distribution: 5 in melon (*Cucumis melo*), 8 in cucumber (*Cucumis sativus*), 8 in watermelon (*Citrullus lanatus*), 7 in bottle gourd (*Lagenaria siceraria*), 7 in wax gourd (*Benincasa hispida*), 13 in moschata pumpkin (*Cucurbita moschata*), and 12 in pumpkin (*Cucurbita maxima*) (Table 1). Predictive analyses of the physicochemical properties of these SRS family proteins revealed significant differences among the 60 Cucurbitaceae SRS proteins. Specifically, the lengths of the encoded amino acids ranged from 211 to 791 aa, the molecular weights ranged from 24,649.34~56,691.2 Da, the theoretical isoelectric points (pI) ranged from 5.98 to 9.19, and the instability coefficients ranged from 37.75 to 66.87. Among these, most of the *SRS* family members were unstable proteins (instability coefficients > 40), and the average hydrophilicity values ranged from −0.876 to −0.165, indicating that most of these proteins are hydrophobic (Table 1). In addition, the identified genes were named according to their positional order on the chromosome rather than following a uniform naming rule.

To clarify the physical location of *SRS* genes in the genomes of these seven cucurbit crops, we mapped the chromosomal distribution of these genes (Figure S1). In melon, 5 *SRS* genes were distributed on chromosomes 1, 3, 4, 7, and 8; in cucumber, 8 *SRS* genes were distributed on chromosomes 1, 2, 3, 4, 5, 6, and 7; in watermelon, 8 *SRS* genes were distributed on chromosomes 1, 2, 3, 4, 5, 6, 7, 8, and 9; in bottle gourd, 7 *SRS* genes were distributed on chromosomes 1, 4, 6, 7, 8, and 10; in wax gourd, 7 *SRS* genes were distributed on chromosomes 1, 2, 3, 5, 7, 9, and 10; in moschata pumpkin, 13 *SRS* genes were distributed on chromosomes 1, 2, 3, 4, 5, 7, 11, 12, 18, 19, and 20; and in pumpkin, 12 *SRS* genes were distributed on chromosomes 1, 2, 3, 4, 5, 7, 11, 12, 18, 16, 19, and 20 (Figure S1). These results indicated that *SRS* genes were dispersed in the genomes of different Cucurbitaceae crops, and there were some differences in distribution among different species.

### 3.2. Analysis of Gene Structure and Conserved Structural Domains of SRS in Cucurbitaceae

**Figure 1:**
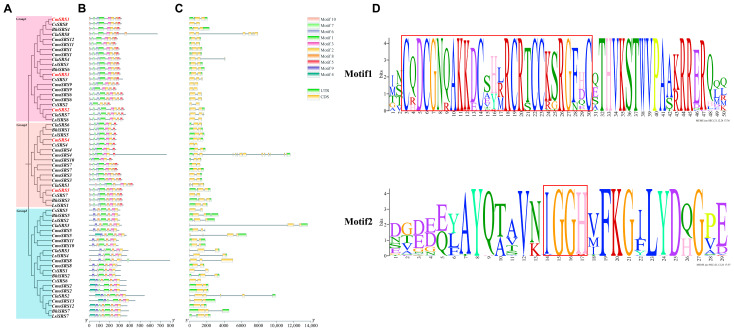
Analysis of gene structure and protein conserved structural domains of *SRS* genes in seven Cucurbitaceae crops. (**A**) Phylogenetic tree of *SRS* genes in Cucurbitaceae. Light red, light brown, and light blue represent the three branches. Red font represents melon *SRS* genes. (**B**) Conserved motifs of cucurbit *SRS* family members. Different colours represent different motifs. (**C**) Cucurbitaceae *SRS* family member gene structures. Green and yellow colours represent UTR and CDS, respectively. (**D**) Cucurbitaceae *SRS* family members Motif1 and Motif2, with red boxes indicating RING and IXGH structural domains.

**Figure 2:**
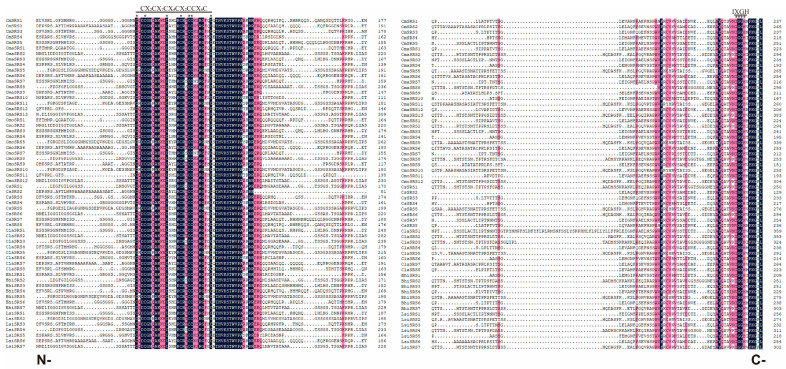
Sequence comparison of SRS family proteins in melon. N- and C- denote the N- and C-termini, respectively, of the *SRS* amino acid sequences of seven Cucurbitaceae crops.

In order to investigate the relationships among *SRS* family members of Cucurbitaceae crops in terms of structure, function, and evolution, a phylogenetic tree was constructed in this study, and the conserved motifs and gene structures of the family members were comprehensively analysed. Phylogenetic tree analysis revealed that the 60 Cucurbitaceae *SRS* family members could be classified into three major branches (Figure 1A), suggesting that they exhibit different evolutionary divergence paths. Further conserved motif analysis revealed that the motif numbers of cucurbit *SRS* family members ranged from 5 to 9 (Figure 1B). All cucurbit SRS proteins contained Motif1 and Motif2, with Motif1 containing the highly conserved RING structural domain characterized by the sequence CX2CX7CX4CX2CCX6C (“X” stands for any amino acid, and the number represents the number of amino acid residues), and Moitf2 containing the highly conserved IXGH structural domain (Figure 1B,D). Amino acid sequence alignment showed that all 60 Cucurbitaceae *SRS* family members contain the RING structural domain at the N-terminus and the IXGH structural domain at the C-terminus. Both structural domains are highly conserved during evolution (Figure 2). Gene structure analysis (Figure 1C) revealed that most of the cucurbit *SRS* family members lacked either the 5′UTR or 3′UTR region. All members, except for *CmoSRS4*, *CmoSRS5*, *ClaSRS2*, *ClaSRS5,* and *ClaSRS8*, contained 1 to 2 introns and 2 to 3 exons. Overall, most of the cucurbit *SRS* genes in the same branch were structurally similar, suggesting a high degree of conservation of gene structure and protein conserved motifs in the cucurbit *SRS* family during evolution. This result provides important clues for understanding the functional divergence and evolution of the *SRS* family in Cucurbitaceae crops.

### 3.3. Evolutionary Analysis of SRS Family Genes in Cucurbitaceae

**Figure 3:**
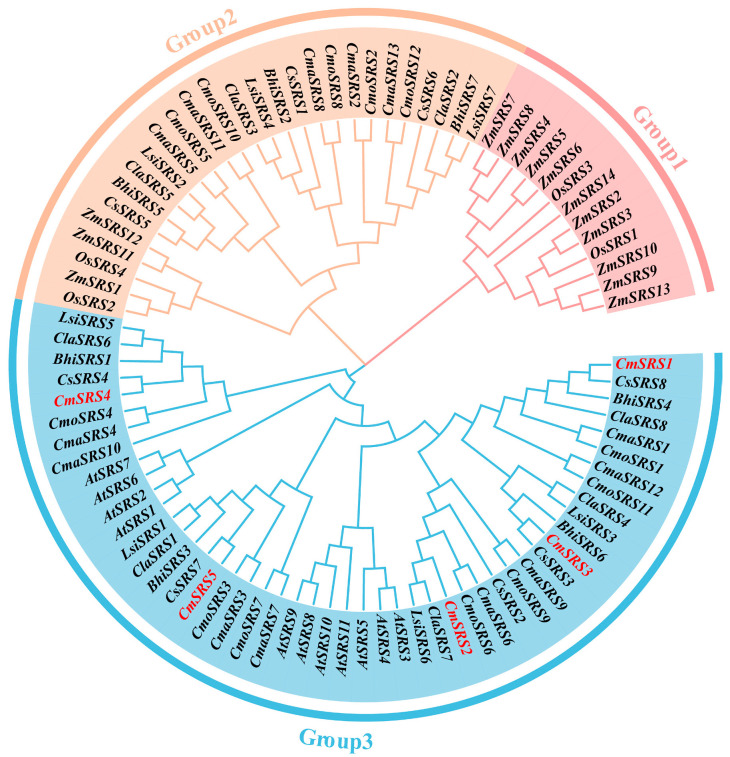
Phylogenetic analysis of *SRS* genes in *Arabidopsis thaliana*, rice, maize, and seven Cucurbitaceae crops. Red font indicates melon *SRS* genes.

To further explore the kinship and evolutionary patterns among the *SRS* genes in Cucurbitaceae, a phylogenetic tree was constructed in this study using 11 *SRS* genes from *Arabidopsis thaliana*, 4 *SRS* genes from rice, 14 *SRS* genes from maize, and 60 *SRS* genes from seven Cucurbitaceae crops via the neighbour-joining method (Figure 3). Based on evolutionary relationships, we classified these genes into three subfamilies (Group 1~3). Among these subfamilies, the Group 3 contained the most members, with 8 *SRS* genes from eight dicotyledonous plants, which contained 5 *CmSRS*, 5 *CsSRS*, 5 *ClaSRS*, 7 *CmaSRS*, 7 *CmoSRS*, *3 LsiSRS*, 4 *BhiSRS*, and 11 *AtSRS*. The Group 2 subfamily contained *SRS* genes from eight crops other than *Arabidopsis thaliana* and melon, with 3 *CsSRS*, 3 *ClaSRS*, 6 *CmaSRS*, 5 *CmoSRS*, 4 *LsiSRS,* 3 *BhiSRS,* 2 *OsSRS*, and 3 *ZmSRS.* The Group 1 subfamily represented only monocotyledonous *SRS* genes, with 11 *ZmSRS* and 2 *OsSRS*. This taxonomic pattern suggests that the SRS proteins in Group 1 and Group 3 may have undergone evolutionary divergence only after the completion of monocotyledonous and dicotyledonous plant differentiation, whereas the Group 2 subfamily may have formed at a much earlier evolutionary stage and display a much wider distribution of members.

**Figure 4:**
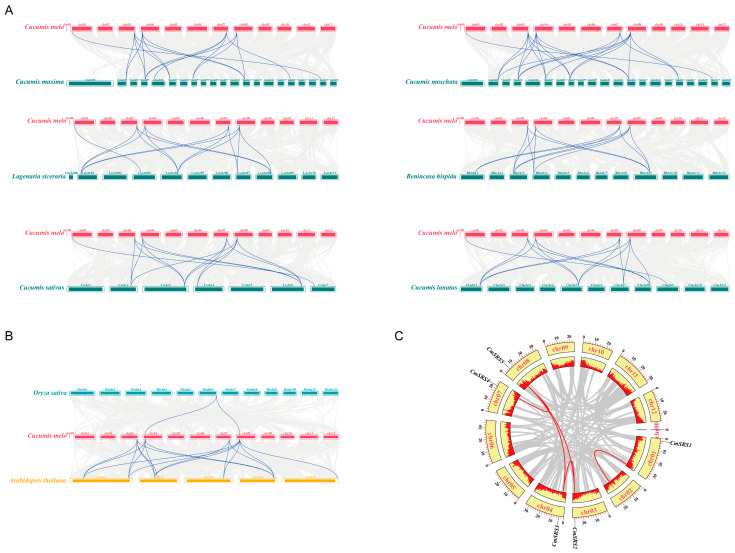
Co-collinearity analysis of *SRS* genes: (**A**) co-collinearity analysis of *SRS* genes in melon and six other Cucurbitaceae crops; (**B**) co-linearity analysis of *SRS* genes in melon, *Arabidopsis thaliana,* and rice; (**C**) *CmSRS* gene duplication analysis. Red lines indicate gene duplication pairs.

To better understand the amplification patterns of the *SRS* family during evolution, covariance analysis of melon and 6 other Cucurbitaceae crops (Figure 4A; Table S3) revealed that melon shares 16, 20, 14, 13, 14, and 14 homologous genes with pumpkin, moschata pumpkin, bottle gourd, wax gourd, cucumber, and watermelon, respectively. These cucurbit crops may have originated from common ancestral genes and retained similar gene structures and functions during evolution. Interestingly, there were covariations between *CmSRS2*, *CmSRS3*, *CmSRS4,* and *CmSRS5* in melon and *SRS* genes of the other six Cucurbitaceae crops, with three pairs of covariations with cucumber, watermelon, wax gourd, and bottle gourd and four pairs with pumpkin. *Arabidopsis thaliana* and rice, as model species for dicotyledonous and monocotyledonous plants, respectively, represent two major taxa of the plant kingdom. Therefore, we performed a combined analysis of melon, *Arabidopsis thaliana*, and rice (Figure 4B). The results showed that there were 18 homologous genes between melon and *Arabidopsis thaliana*, whereas there was only 1 homologous gene with rice. This difference suggests that monocotyledons and dicotyledons may not share a large number of homologous genes prior to their divergence, which further emphasises the independent evolutionary pathways of the *SRS* family in different plant taxa. Gene duplication events are an important mechanism for gene family amplification, usually caused by genome-wide duplications or segmental duplications. Gene duplication significantly affects the diversity and conservation of gene functions. To explore gene duplication events in *SRS* family members in melon, we performed within-species covariance analysis in melon (Figure 4C). The results revealed four pairs of duplicated genes in melon: *CmSRS2-CmSRS3*, *CmSRS5-CmSRS4*, *CmSRS5-CmSRS3*, and *CmSRS5-CmSRS2*. *CmSRS5* was involved in three segmental duplications, while *CmSRS2* and *CmSRS3* were each involved in two segmental duplications. These results suggest that segmental duplication events may be the main driver of *SRS* family amplification in melon. They also provide the basis for the generation and diversity of new gene functions.

### 3.4. Analysis of Cis-Acting Elements in the Promoter Sequence of Melon SRS Genes

**Figure 5:**
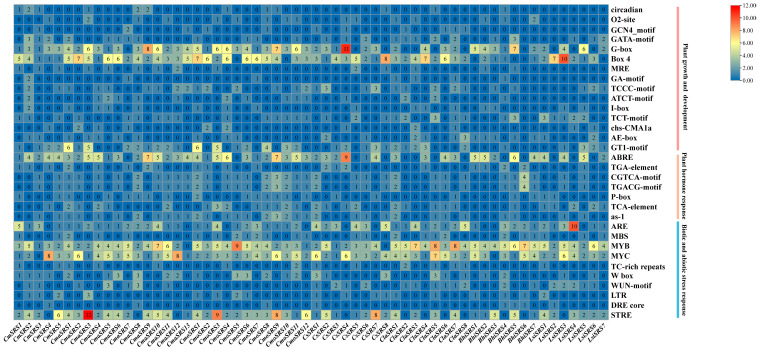
*Cis*-acting elements in the promoters of *SRS* genes in seven Cucurbitaceae crops. Colours indicate the number of different *cis*-acting elements. Numbers indicate the statistical number of *cis*-acting elements.

Plants respond to biotic and abiotic stresses through complex regulatory mechanisms, many of which depend on *cis*-acting elements in gene promoter regions. To investigate the transcriptional regulation of the *SRS* genes and its potential functions, we predicted *cis*-acting elements in the promoter regions of 60 Cucurbitaceae *SRS* genes. We found core elements such as the CAAT-box and the TATA-box in all *SRS* genes, as well as 32 different *cis*-acting elements related to biotic and abiotic stress response, plant growth and development, and phytohormone response (Figure 5). Among them, 15 elements were related to growth and development, including circadian, O2-stie and GCN4_motif; 10 elements were related to biotic and abiotic stresses, including ARE, MBS, MYB, MYB, and MBS; 10 elements were related to drought response, including MYB, MBS, and MYB; 10 elements were related to plant growth and development; 10 elements were related to plant growth and development, including MYB, MYB, and MBS; 10 elements were related to plant growth and development, such as MYB; and 10 other elements and the action elements were related to phytohormone response, including ABRE (abscisic acid response element), TGA- element (growth hormone response element), CGTCA-motif, TGACG-motif (jasmonic acid response element), P-box (gibberellin response element), and TCA-element (salicylic acid response element).

Among the growth and development-related *cis*-acting elements, the light-responsive elements G-box and Box4 were widely distributed in most cucurbit *SRS* genes and were most abundant in *CsSRS4* and *LsiSRS3*, respectively. Among the phytohormone-related acting elements, the ABRE was most abundantly distributed among the cucurbit *SRS* genes, especially in the melon (*CmSRS*) family, where all members contained this element. Among the biotic and abiotic stress response elements, the drought response element (MYC) appeared in 58 Cucurbitaceae crop *SRS* genes, whereas the defence and stress response element (MYB) was also widely distributed in this family of genes. Among *the CmSRS* genes of melon, all members except *CmSRS3* contained MYC elements, and all members contained MYB elements, suggesting that *the CmSRS* genes may play an important role in drought and defence response in melon. In summary, the *SRS* genes of seven cucurbit crops exhibit important functions in physiological processes such as plant growth and development, hormone signal transduction, and stress response. The diversity of their *cis*-acting elements provides a basis for their regulation in a variety of biological processes.

### 3.5. Tissue-Specific Expression Analysis of SRS Genes in Melon

**Figure 6:**
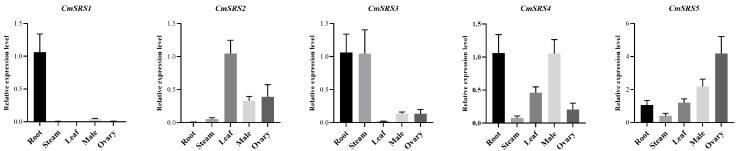
Tissue-specific expression analysis of *SRS* genes in melon. Values are mean ± SD of three biological replicates.

In order to investigate the expression patterns of melon *SRS* family members in different tissues, we analysed the tissues of roots, stems, leaves, stamens, and ovaries of melon by qRT-PCR. The results (Figure 6) showed that the expression levels of the five *CmSRS* family members in different tissues differed significantly. Among them, *CmSRS1*, *CmSRS3* and *CmSRS4* showed higher expression levels in the roots, whereas *CmSRS2* and *CmSRS5* showed higher expression in the leaves and ovaries, respectively. The expression of *CmSRS1* was mainly observed in the roots, and expression was very low in the other tissues. *CmSRS3* expression in the stems was significantly higher than that of the other four *CmSRS* members. These results suggest that melon *SRS* genes display tissue-specific expression patterns during their growth and development, which may play an important role in regulating the organ development and functional differentiation of melon.

### 3.6. Expression Analysis of SRS Family Genes in Melon Under Drought and Salt Stresses

**Figure 7:**
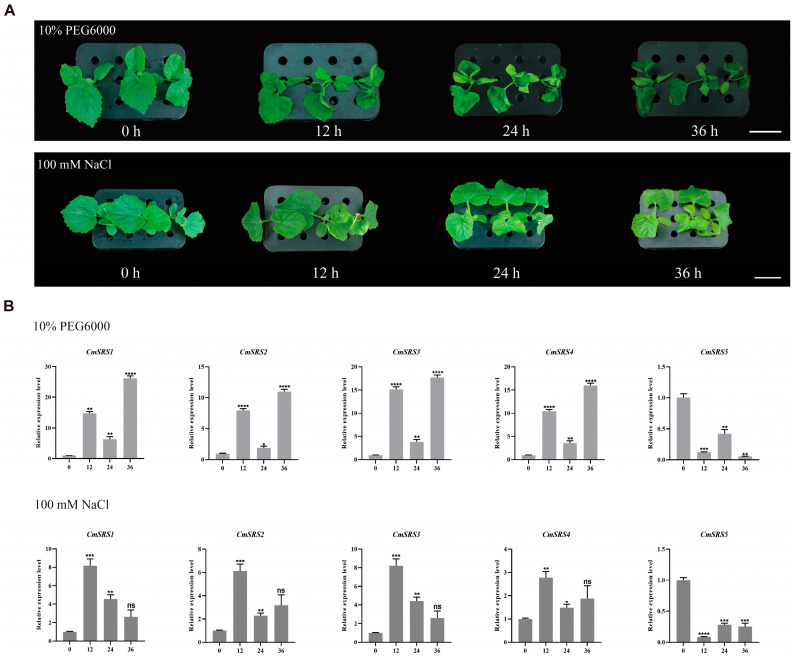
Expression analysis of melon *SRS* genes under drought and salt stress. (**A**) Phenotypic observations of melon after 0, 12, 24, and 36 h of 20% PEG6000 treatment. (**B**) Expression analysis of 5 *CmSRS* genes under drought stress. Values are mean ± SD of three biological replicates. Asterisks indicate significant differences between 0 h and other time points (ns: no significance; * *p* < 0.05; ** *p* < 0.01; *** *p* < 0.001; **** *p* < 0.0001).

Drought and salt stress are two of the most important environmental factors affecting plant growth and development, as they alter the crop’s access to nutrients and thus affect the final yield and quality [33,34,35]. In soybean, the *GmSRS18* gene has been shown to be a negative regulator in response to drought stress [1]. To investigate whether melon *SRS* genes were induced by drought and salt stress, melon roots were treated with 20% PEG6000 and 100 mM NaCl (Figure 7A). The expression was analysed by qRT-PCR after 0, 12, 24, and 36 h treatments (Figure 7B). The results showed that the five *CmSRS* genes responded to both drought and salt stresses. Under drought stress, the expression of *CmSRS1/2/3/4* showed an increasing trend at 12 h, declined at 24 h, and then increased again to the highest value at 36 h. In contrast, the expression pattern of *CmSRS5* was the opposite, with the lowest expression at 36 h. Under salt stress, the expression levels of *CmSRS1/2/3/4* all peaked at 12 h and showed a decreasing trend at 24 h. However, *CmSRS2* and *CmSRS4* showed an increasing trend at 36 h, whereas *CmSRS1* and *CmSRS3* continued to decrease. The expression pattern of *CmSRS5* was in the opposite direction, with the lowest expression at 12 h. In addition, it was observed that melon already showed wilting after 12 h of treatment, suggesting that *CmSRS* genes may play an important regulatory role in melon response to drought and salt stress.

### 3.7. Expression Analysis of SRS Family Genes in Melon Under Biotic Stresses

**Figure 8:**
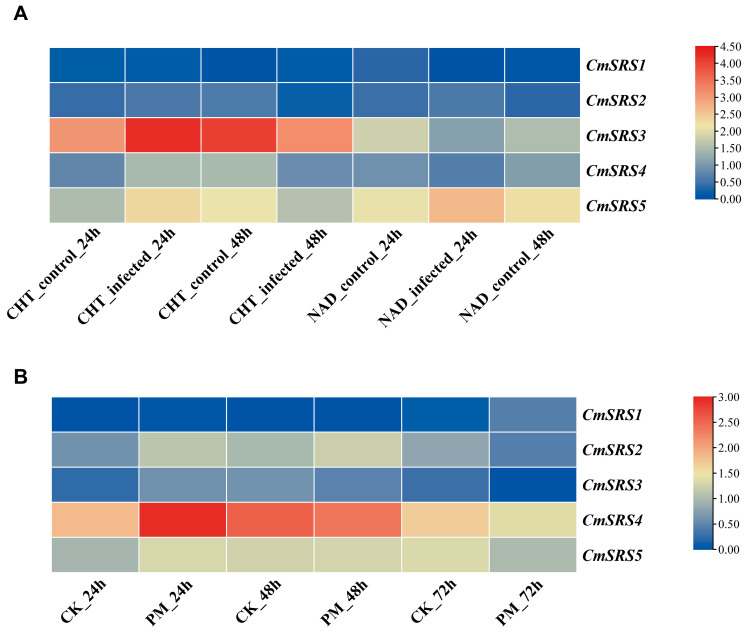
Expression analysis of *SRS* genes in melon upon biotic stresses. (**A**) Expression analysis of *CmSRS* genes in CHT and NAD varieties infested with *Fusarium spinosum* for 24 h and 48 h. (**B**) Expression analysis of *CmSRS* genes infested with powdery mildew fungus for 24 h, 48 h and 72 h.

Powdery mildew and wilt are major fungal diseases in melon production, which seriously affect the growth, development, yield, and quality of melon. To investigate the response patterns of melon *SRS* genes under biotic stresses (wilt and powdery mildew), the expression responses of the disease-resistant melon variety NAD and the susceptible variety Charentais-T (CHT) to wilt and that of the variety Rochet to powdery mildew were analysed using the NCBI SRA public transcriptome database (Figure 8). After 24 h of infection with *Fusarium spinosum*, the expression of all *CmSRS* genes increased in the susceptible variety CHT, except for a slight decrease noted in the expression of *CmSRS1*, with a greater increase in *CmSRS3*. The expression levels of *CmSRS2* and *CmSRS5* increased, while the expression levels of *CmSRS1*, *CmSRS3*, and *CmSRS4* declined in the disease-resistant variety NDA. After 48 h of infection, the expression levels of all *CmSRS* genes decreased in the susceptible variety CHT, except for *CmSRS1*, in which the expression level of *CmSRS3* decreased to a greater extent. The expression levels of the five *CmSRS* genes increased in the disease-resistant variety NAD. The expression levels of all five *CmSRS* family genes increased 24 h after infestation with powdery mildew pathogens. At 48 h post infestation, the expression of *CmSRS2* and *CmSRS5* increased. Additionally, the expression of *CmSRS3* and *CmSRS4* decreased, while the expression of *CmSRS1* remained unchanged. At 72 h post infestation, the expression levels of all five *CmSRS* genes decreased. These results indicated that melon *SRS* genes showed different expression patterns under wilt and powdery mildew stresses, and significant differences existed between resistant and susceptible varieties.

### 3.8. Analysis of Subcellular Localization of SRS Family Genes in Melon

**Figure 9:**
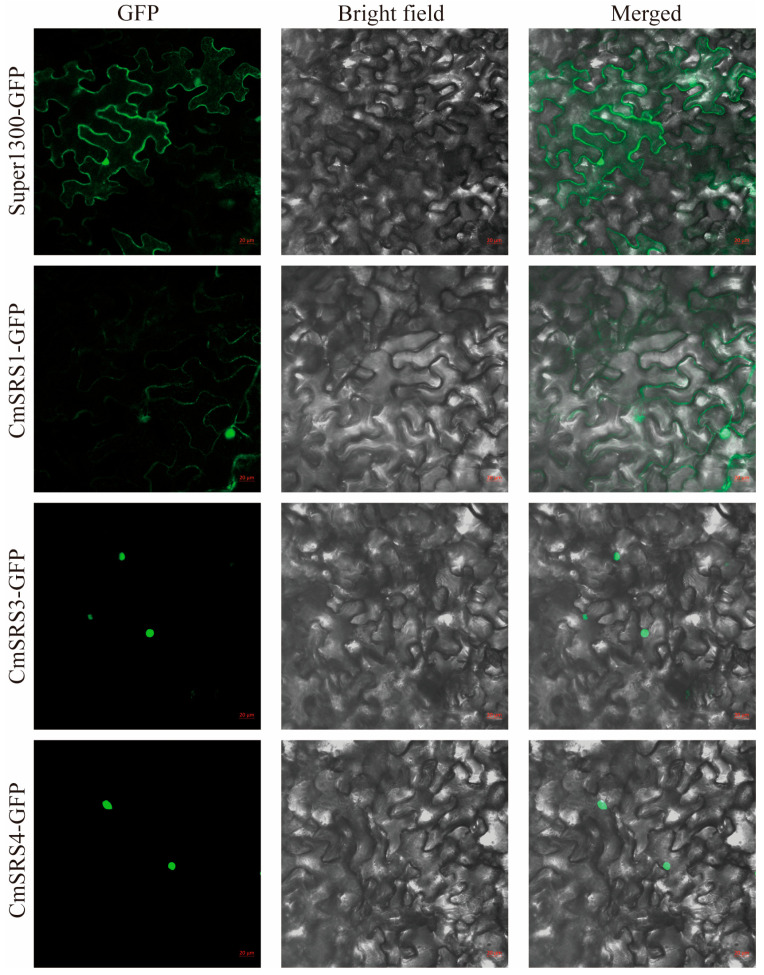
Analysis of subcellular localization of CmSRS protein. Subcellular localization of CmSRS protein fused to GFP and transiently expressed in *Nicotiana benthamiana* leaf cells was observed by laser scanning confocal microscopy, using Super1300-GFP empty vector as a control. Bars  =  20 μm.

Based on these results, we selected *CmSRS1*, *CmSRS3*, and *CmSRS4*, which are highly expressed in roots, and analysed their protein functions. To detect their subcellular localization, we constructed CmSRS proteins fused with green fluorescent protein (GFP) and transiently expressed them in *Nicotiana benthamiana* leaves (Figure 9). The results showed that GFP fluorescent signals were observed only in the nucleus for *CmSRS3* and *CmSRS4*, which was caused by the nuclear input of transcription factors contributing to transcriptional activity. CmSRS1-GFP fluorescent signals were observed in both the nucleus and the cell membrane, with a predominant nuclear localisation, which is likely regulated post-translationally.

### 3.9. Interaction Network and Structure Prediction of SRS Family Proteins in Melon

**Figure 10:**
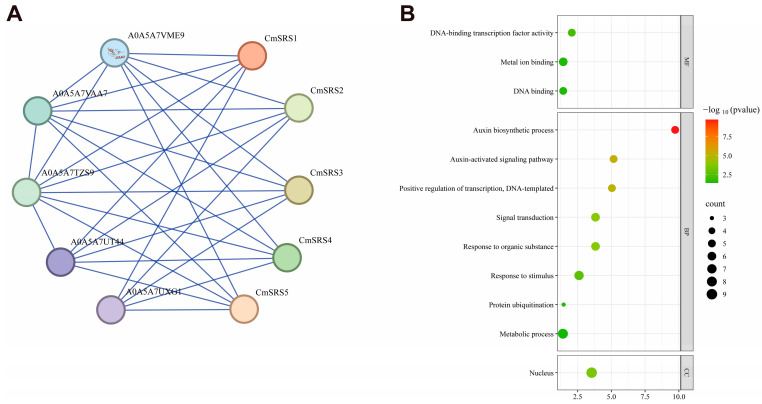
Predicted interactions of melon SRS proteins: (**A**) predicted interactions of five CmSRS proteins; (**B**) GO enrichment analysis.

**Figure 11:**
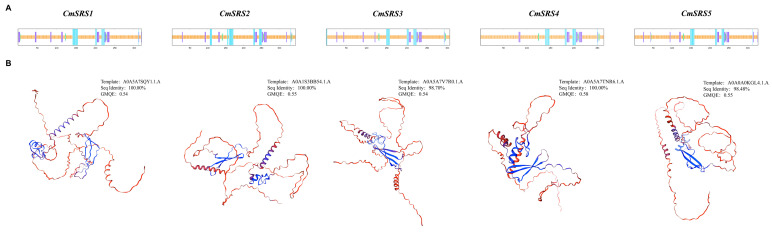
Predicted structure of melon SRS proteins: (**A**) secondary structure of five CmSRS proteins; (**B**) tertiary structure of five CmSRS proteins.

To further explore the biological functions of SRS proteins in melon, we searched and analysed their potential interacting proteins using the STRING database (Version 12.0). Based on the prediction, all five CmSRS proteins were found to interact with A0A5A7TZS9 (ethylene-inducible protein), A0A5A7VAA7 (calcium-binding EF-hand family protein), A0A5A7VME9 (RING-type E3 ubiquitinyltransferase), A0A5A7UT44 (F-box protein PP2-B10 analogue), and A0A5A7UXG1 (F-box protein PP2-B10 analogue isoform X1) (Figure 10A; Table S4). These proteins are involved in lutein biosynthesis (Figure 10B), implying that CmSRS proteins may have an important role in metabolic regulation in melon. Further protein secondary structure analysis showed (Figure 11A; Table S5) that the secondary structure of melon *CmSRS* family member proteins consisted of an α-helix, β-turn, an extended strand, and a random coil. The prediction results showed that a large number of irregular coils with a small number of α-helices and extended strands, along with a very small number of β-turns, were present in these five CmSRS proteins. In addition, tertiary structure analysis of melon SRS proteins showed (Figure 11B) that the three-dimensional structures of this family of proteins are highly similar, which may be related to their functional conservation.

## 4. Discussion

Thus far, the *SRS* genes have been identified in a variety of plants, including *Arabidopsis thaliana* [6], rice [2], cotton [19], maize [36], and cucumber [37], among others. Among them, the *SRS* genes in *Arabidopsis thaliana* are the most intensively and comprehensively studied, involving several biological processes such as root formation [12], leaf development [38], and floral organ development [39]. However, among cucurbit crops, the *SRS* genes have only been identified in cucumber. Therefore, this study systematically identified *SRS* genes in seven cucurbit crops via bioinformatics and focused on the expression pattern of *SRS* genes in melon to fill the gap in the study of *SRS* genes in Cucurbitaceae crops.

In this study, 5, 8, 8, 7, 7, 13, and 12 *SRS* genes were identified in seven Cucurbitaceae crops including melon, cucumber, watermelon, bottle gourd, wax gourd, moschata pumpkin, and pumpkin, respectively. These genes were randomly distributed on the chromosomes. All *CmSRS* family members contained the RING structural domains (CX2CX7CX4CX2C2X6C) and the IXGH structural domains, which is in agreement with the findings in *Arabidopsis thaliana* [6]. These results indicate the conservation of SRS proteins in these seven Cucurbitaceae crops. However, the parameters of the protein physicochemical properties such as amino acid number, isoelectric point, and instability coefficient varied among crops, which may be related to the structural diversity of *SRS* genes. Gene structure provides clues to the diversity of gene functions and plays a crucial role in the evolution of gene families [40]. It has been reported that members of the *CqSRS* family contain two to five exons, and genes within the same subfamily exhibit similar gene structures [41]. To explore the structural diversity of *SRS* genes in seven Cucurbitaceae crops, we analysed their gene structures and protein conserved motifs and found that 60 cucurbit *SRS* genes had two to three exons and one to two introns. In addition, all contain Motif1, with a RING structural domain, and Motif2, with an IXGH structural domain. We also found that genes in the same subfamily display similar protein motifs and gene structures, suggesting that they may have similar functions.

To better understand the evolutionary relationships of the *SRS* family, the phylogenies of seven Cucurbitaceae crops were compared with *Arabidopsis thaliana*, rice and maize. It was found that the genes of the *SRS* family in monocotyledonous and dicotyledonous plants belonged to different branches, which is similar to the findings in cucumber [37]. Gene duplication events play an important role in genome amplification and gene functional diversity [42]. In order to investigate the amplification pattern of *SRS* genes in seven Cucurbitaceae crops during the evolutionary process, by covariance analysis, we found that melon possesses 16, 20, 14, 13, 14, and 14 homologous genes with pumpkin, moschata pumpkin, bottle gourd, wax gourd, cucumber, and watermelon, respectively. Interestingly, all of the *CmSRS2/3/4/5* in melon are covaried with *SRS* genes in six other Cucurbitaceae crops, such as cucumber, watermelon, wax gourd, and bottle gourd with three pairs of co-linear genes, and pumpkin with four pairs of co-linear genes. In addition, four pairs of duplicated genes were identified in *CmSRS* family members, of which *CmSRS5* was involved in three segmental duplications, and *CmSRS2* and *CmSRS3* were each involved in two segmental duplications, suggesting that segmental duplication events may be the main drivers of gene amplification in the *CmSRS* family.

Gene promoter region action elements play an important role in plant defence against biotic and abiotic stresses [43]. A large number of elements involved in stress response and growth and development were found in the *ZmSRS* family genes of maize [36]. This suggests that *SRS* genes may play a role in pathways related to plant stress response mechanisms. The seven Cucurbitaceae *SRS* genes contain low temperature response elements (LTR), drought response elements (MBS and MYC), and defence and stress response elements (TC-rich repeats, MYB, and W-box), as well as a wide range of components related to *SRS* transcription factors. These factors are involved in the development of plant organs and tissues by regulating the synthesis and signal transduction of a variety of plant hormones. In *Arabidopsis thaliana*, *AtLRP1* regulates root development [14], and *AtSTY1* is involved in leaf and flower development [44,45,46]. In this study, the expression pattern of melon *SRS* family genes in different tissues was analysed by qRT-PCR. *CmSRS1*, *CmSRS3,* and *CmSRS4* were highly expressed in the roots, whereas *CmSRS2* and *CmSRS5* were highly expressed in the leaves and ovaries, respectively. This suggests that the *CmSRS* genes are tissue-specific in the growth and development of different organs in melon. Plants are affected by biotic and abiotic factors during the growth cycle. In *Arabidopsis thaliana*, *SRS5* gene expression can be significantly upregulated by pathogen induction, suggesting that *AtSRS5* may be involved in regulating the immune response of plants [47]. In the present study, we found that the expression of five *CmSRS* genes was elevated after 48 h of infestation by NAD in wilt-resistant varieties and after 24 h of infestation by powdery mildew pathogens through the response of melon to wilt and powdery mildew diseases. In addition, abiotic stresses also induce the expression of *SRS* genes. For example, *GmSRS18* in soybean is induced by drought, NaCl, and exogenous ABA. Its overexpression increases the sensitivity of transgenic *Arabidopsis thaliana* to drought and salt stresses [1]. We further investigated the expression pattern of *CmSRS* genes under drought stress. It is noteworthy that *CmSRS1/2/3/4* showed an increasing trend at 12 h under drought stress, declined at 24 h, and increased again to its highest levels at 36 h, whereas under salt stress, *CmSRS1/2/3/4* peaked at 12 h and declined at 24 h. These results indicate that the *CmSRS* genes are diverse in their response to drought and salt stress. Combined with the prediction of promoter *cis*-acting elements, we found that the all *CmSRS* genes contain drought-responsive elements (MYC), as well as stress-responsive elements (MYB). This suggests that melon *SRS* genes may play an important role in abiotic stress response.

## 5. Conclusions

In summary, in this study, a total of 60 *SRS* genes were identified from seven Cucurbitaceae crops. Their chromosomal distribution, protein physicochemical properties, gene structure and protein conserved motifs, conserved structural domains, evolutionary relationships, gene duplications, and *cis*-acting elements were systematically analysed. The results showed that the *SRS* genes of cucurbit crops were classified into three subfamilies. five *SRS* genes of melon and six other Cucurbitaceae crop *SRS* genes were duplicated, thus expanding the *SRS* gene family of Cucurbitaceae crops. In addition, *CmSRS2/3/4/5* exhibited duplication events within melon species. Gene structure and protein conserved motif results indicated that the same subfamily displays similar conserved motifs and gene structures. Analysis of promoter *cis*-regulatory elements indicated that these *SRS* genes may be involved in hormone response, growth and development, and biotic and abiotic stress response in plants. In addition, most of the *CmSRS* genes are expressed in the roots, and a few are expressed in the leaves and ovaries. Drought and salt stresses often exacerbate diseases such as powdery mildew and wilt by indirectly weakening the physiological condition of melon plants and altering the rhizosphere environment. Transcriptome analysis of melon roots subjected to 20% PEG6000, 100 mM NaCl, wilt, and powdery mildew revealed that *CmSRS* gene expression was responsive to all four stress conditions. The subcellular localization and structure of CmSRS proteins were further investigated. This study provides insights into the functions of *SRS* genes in the growth and development of cucurbit crops and stress response, laying a solid foundation for subsequent functional validation and application studies.

## Figures and Tables

**Table 1:** Physicochemical properties of SRS proteins in Cucurbitaceae.

Gene Name	Gene ID	AA	MW(Da)	pI	Instability Index	Aliphatic Index	Grand Average of Hydropathicity
*CmSRS1*	*MELO3C018686.1*	320	35,379.07	6.55	55.97	60.62	−0.733
*CmSRS2*	*MELO3C010984.1*	347	37,430.36	6.45	57.79	53.08	−0.615
*CmSRS3*	*MELO3C003781.1*	307	34,469	8.04	52.18	54.92	−0.821
*CmSRS4*	*MELO3C027042.1*	265	28,899.03	5.98	60.84	46.75	−0.709
*CmSRS5*	*MELO3C007877.1*	328	35,598.99	6.96	54.16	50	−0.769
*CmaSRS1*	*CmaCh01G019390.1*	297	32,894.42	6.36	44.45	54.85	−0.724
*CmaSRS2*	*CmaCh02G012730.1*	363	37,868.88	7.14	46.07	67.77	−0.398
*CmaSRS3*	*CmaCh03G007080.1*	316	34,060.38	6.52	54.28	52.22	−0.701
*CmaSRS4*	*CmaCh04G007690.1*	263	28,771.27	6.99	52.33	56.05	−0.573
*CmaSRS5*	*CmaCh04G013270.1*	292	30,939.92	9.14	42.92	71.13	−0.328
*CmaSRS6*	*CmaCh05G002810.1*	337	36,312.15	6.4	58.68	52.02	−0.575
*CmaSRS7*	*CmaCh07G004520.1*	286	31,221.31	7.09	57.93	52.94	−0.739
*CmaSRS8*	*CmaCh11G008840.1*	791	84,224.85	5.73	46.89	68.31	−0.343
*CmaSRS9*	*CmaCh12G004300.1*	297	32,178.62	7.2	56.66	54.34	−0.569
*CmaSRS10*	*CmaCh16G006400.1*	229	24,649.34	6.74	51.25	54.59	−0.593
*CmaSRS11*	*CmaCh18G008580.1*	288	30,660.29	9.35	48.71	61.01	−0.518
*CmaSRS12*	*CmaCh19G002830.1*	280	31,479.16	8.3	47.86	61.25	−0.695
*CmaSRS13*	*CmaCh20G001080.1*	451	46,311.77	6.05	38.83	65.23	−0.393
*CmoSRS1*	*CmoCh01G020010.1*	298	33,041.58	6.55	46.82	55	−0.751
*CmoSRS2*	*CmoCh02G013150.1*	364	38,054.02	7.19	46.54	67.58	−0.404
*CmoSRS3*	*CmoCh03G007340.1*	323	34,976.26	6.36	53.38	50.77	−0.757
*CmoSRS4*	*CmoCh04G008220.1*	757	84,845.82	8.74	46.24	76.06	−0.226
*CmoSRS5*	*CmoCh04G014000.1*	366	39,658.77	9.01	46.59	71.69	−0.386
*CmoSRS6*	*CmoCh05G002870.1*	337	36,308.17	6.4	58.6	52.02	−0.578
*CmoSRS7*	*CmoCh07G004500.1*	282	30,684.85	7.06	56.94	54.4	−0.685
*CmoSRS8*	*CmoCh11G008930.1*	313	31,704.21	9.06	39.01	57.7	−0.322
*CmoSRS9*	*CmoCh12G003810.1*	267	28,731.75	8.62	58.69	50.6	−0.567
*CmoSRS10*	*CmoCh18G008640.1*	286	30,427.01	9.23	45.66	63.15	−0.484
*CmoSRS11*	*CmoCh19G003110.1*	267	30,091.45	6.23	45.87	57.68	−0.731
*CmoSRS12*	*CmoCh20G001160.1*	373	38,508.3	6.88	36.08	64.91	−0.406
*CsSRS1*	*CsaV3_1G043360.1*	307	31,146.62	8.9	41.44	58.18	−0.338
*CsSRS2*	*CsaV3_2G029220.1*	211	21,977.57	6.87	44.57	57.63	−0.225
*CsSRS3*	*CsaV3_3G002100.1*	388	43,201.45	8.93	49.09	67.16	−0.865
*CsSRS4*	*CsaV3_3G045570.1*	329	37,236.52	9	48.97	60.09	−0.806
*CsSRS5*	*CsaV3_4G005360.1*	263	28,682.78	6.24	61.27	45.59	−0.751
*CsSRS6*	*CsaV3_5G036780.1*	303	32,073.74	8.89	41.15	62.21	−0.493
*CsSRS7*	*CsaV3_6G039030.1*	365	37,662.42	7.15	37.75	61.21	−0.417
*CsSRS8*	*CsaV3_6G046800.1*	331	35,897.29	7	54.07	50.45	−0.769
*CsSRS9*	*CsaV3_7G006060.1*	317	35,221.94	7.13	53.15	60.88	−0.736
*ClaSRS1*	*Cla97C01G019470.2*	435	48,014.42	8.72	54.09	56.34	−0.63
*ClaSRS2*	*Cla97C02G048970.2*	542	56,691.2	8.62	47.87	73.12	−0.232
*ClaSRS3*	*Cla97C03G062920.2*	382	38,886.5	9.19	43.25	65.99	−0.183
*ClaSRS4*	*Cla97C05G099960.2*	311	34,917.29	6.53	50.85	49.52	−0.876
*ClaSRS5*	*Cla97C05G109100.2*	318	33,822.69	8.95	43.59	62.01	−0.533
*ClaSRS6*	*Cla97C07G135860.2*	275	29,953.39	6.65	54.08	54.65	−0.602
*ClaSRS7*	*Cla97C08G158100.2*	359	39,151.43	6.61	59.13	52.65	−0.655
*ClaSRS8*	*Cla97C09G176200.2*	669	76,183.14	6.04	66.87	72.86	−0.588
*BhiSRS1*	*Bhi01M001886*	265	28,659.96	6.08	55.08	50.08	−0.581
*BhiSRS2*	*Bhi02M001250*	308	30,990.5	8.96	43.1	62.47	−0.264
*BhiSRS3*	*Bhi03M000673*	328	35,617.15	6.96	54.47	50.91	−0.747
*BhiSRS4*	*Bhi05M000625*	306	34,206.05	6.49	39.03	61.76	−0.665
*BhiSRS5*	*Bhi07M001030*	302	31,942.66	8.89	44.19	64.67	−0.463
*BhiSRS6*	*Bhi09M001884*	307	34,150.55	6.89	52.03	55.24	−0.817
*BhiSRS7*	*Bhi10M000200*	387	40,243.5	7.24	43.53	69.59	−0.352
*LsiSRS1*	*Lsi01G006310.1*	335	36,537.99	6.88	54.6	47.52	−0.808
*LsiSRS2*	*Lsi04G000260.1*	301	31,654.32	8.83	43.82	63.59	−0.468
*LsiSRS3*	*Lsi04G017130.1*	299	33,410.68	6.55	48	49.87	−0.857
*LsiSRS4*	*Lsi06G005760.1*	369	37,719.13	8.5	45.78	63.31	−0.165
*LsiSRS5*	*Lsi07G002300.1*	273	29,457.71	6.3	53.85	50.81	−0.549
*LsiSRS6*	*Lsi08G013510.1*	336	36,292.11	6.66	60.64	52.5	−0.622
*LsiSRS7*	*Lsi10G010870.1*	372	38,375.3	8.36	42.65	66.64	−0.376

## Data Availability

The datasets supporting the conclusions of this article are included within the article and its additional files. Genomic sequences and gene annotation information of *Cucumis melo*, *Cucumis sativus*, *Citrullus lanatus, Lagenaria siceraria, Benincasa hispida*, *Cucurbita moschata,* and *Cucurbita maxima* were downloaded from http://www.cucurbitgenomics.org/. (accessed on 15 September 2024) Genomic sequences and gene annotation information of *Arabidopsis thaliana*, rice, and maize were downloaded from https://plants.ensembl.org/ (accessed on 15 September 2024).

## Supplementary Materials

The following supporting information can be downloaded at: https://www.mdpi.com/article/10.3390/biology14070891/s1. Supplemental Figure S1. Chromosomal distribution of *SRS* genes in seven cucurbit crops. Supplemental Table S1. RT-qPCR primers for *CmSRS* genes. Supplemental Table S2. Subcellular localization primers for *CmSRS* genes. Supplemental Table S3. Orthologous relationships of *SRS* genes in melon and six other cucurbit species. Supplemental Table S4. CmSRS protein interaction prediction information. Supplemental Table S5. Prediction of secondary structure of *SRS* gene family proteins in melon.

## Abbreviations

aa	Amino Acid
ABA	Abscisic Acid
bp	Base Pair
BP	Biological Process
CC	Cellular Component
CDD	Conserved Domain Database
cDNA	Complementary DNA
CDS	Coding Sequence
CHT	Charentais-T
Da	Dalton
GA	Gibberellic Acid
HMM	Hidden Markov Model
IAA	Indole-3-Acetic Acid
LRP1	Lateral Root Primordium1
MeJA	Methyl Jasmonate
MEME	Motif-Based Sequence Analysis Tools
MF	Molecular Function
qRT-PCR	Quantitative Real-Time PCR
SA	Salicylic Acid
SHI	Short Internodes
